# Exenatide Delays the Progression of Nonalcoholic Fatty Liver Disease in C57BL/6 Mice, Which May Involve Inhibition of the NLRP3 Inflammasome through the Mitophagy Pathway

**DOI:** 10.1155/2018/1864307

**Published:** 2018-04-15

**Authors:** Ning Shao, Xin-Yang Yu, Xue-Fei Ma, Wen-Jian Lin, Ming Hao, Hong-Yu Kuang

**Affiliations:** Department of Endocrinology, The First Affiliated Hospital of Harbin Medical University, Harbin, Heilongjiang 150001, China

## Abstract

**Objective:**

This study is aimed at investigating whether exenatide (Exe) delays the progression of nonalcoholic fatty liver disease (NAFLD) in C57BL/6 mice by targeting the NLRP3 inflammasome through the autophagy/mitophagy pathway.

**Methods:**

Thirty male C57BL/6 mice were randomly divided into three groups: control group (*n* = 10), model group (*n* = 10), and Exe (exenatide) group (*n* = 10). Mouse models of NAFLD and diabetes were established using a high-fat diet and streptozocin.

**Results:**

The levels of fasting blood glucose (FBG), total cholesterol (TC), and triglyceride (TG) in the serum were significantly reduced after Exe treatment. The body weight, liver weight/body weight, and number of lipid droplets in the liver significantly decreased in Exe-treated mice. Treatment with Exe markedly reduced the levels of liver lipids, malondialdehyde (MDA), and alanine aminotransferase (ALT) in serum and livers. The number of autophagosomes increased significantly in the Exe group. The expression of LC3A/B-II/I, Beclin-1, Parkin, and BNIP3L increased significantly, whereas NLRP3 and IL-1*β* proteins were suppressed after Exe treatment.

**Conclusion:**

We successfully established a mouse model of NAFLD and diabetes. Exe may reduce oxidative stress injury and inhibit the NLRP3 inflammasome by enhancing the autophagy/mitophagy pathway in liver, which has a protective effect on the liver in NAFLD and diabetes in C57BL/6 mice.

## 1. Introduction

Increasing evidence has indicated a complex interplay between type 2 diabetes mellitus (T2DM) and nonalcoholic fatty liver disease (NAFLD) [1]. NAFLD incorporates a spectrum of pathologies that range from simple steatosis to nonalcoholic steatohepatitis (NASH) to fibrosis and cirrhosis, with an increased risk of hepatocellular carcinoma. Epidemiologic data suggest that diabetes is present in 33% to 50% of all patients with NAFLD and in up to 60–80% of T2DM patients with NAFLD [2]. In addition, both conditions are associated with a high risk of developing cardiovascular disease [2]. In these patients, excessive lipid metabolites accumulate in hepatocytes, which disrupts the balance between the oxidative and antioxidative capacities of these hepatocytes [3, 4]. Lipotoxicity may provoke hepatocyte injury by inducing mitochondrial dysfunction and endoplasmic reticulum stress [5]. Day and James [6] presented a classic “two-hit” hypothesis to describe the pathogenesis of NAFLD whereby insulin resistance (IR) contributes to steatosis (first hit), which sensitizes the liver to oxidative stress (second hit), resulting in inflammation, fibrosis, and necrosis. The latest theory is the “multivariate parallel collision theory,” which states that NAFLD is a process that involves the accumulation of hepatotoxic damage [7]. In line with the hepatocyte-derived hits, inflammatory signals from the oxidative stress can further exacerbate hepatic inflammation.

The NLRP3 inflammasome is a multiprotein complex that recognizes various pathogen-associated molecular patterns (PAMPs) and damage-associated molecular patterns (DAMPs). The key components of a functional NLRP3 inflammasome include nucleotide-binding oligomerization domain- (NOD-) like receptor P3 (NLRP3), the adaptor protein apoptosis-associated speck-like protein containing a caspase recruitment domain (ASC), and serine protease caspase-1 (Casp1)[8]. Upon detecting endogenous or exogenous danger signals, NLRP3 recruits ASC and procaspase-1, resulting in caspase-1 activation, which further cleaves prointerleukin- (IL-) 1*β* and pro-IL-18 to yield their active forms and leads to chronic inflammation in metabolic diseases. The world-renowned research group of Jürg Tschopp found that mitochondrial reactive oxygen source (ROS) is a key signal in regulating the activation of the NLRP3 inflammasome and that autophagy/mitophagy may regulate the quality of mitochondria by removing damaged ones to prevent ROS-induced NLRP3 inflammasome activation in THP1 cells [9]. A previous study has demonstrated that the depletion of the autophagic protein microtubule-associated protein-1 light chain 3 (LC3) B and Beclin-1 enhances both caspase-1 activation and the secretion of interleukin-1*β* and interleukin-18 [10]. Thus, autophagy negatively regulates the activation of the NLRP3 inflammasome by preserving mitochondrial integrity. NLRP3 is a key factor in the development of NAFLD to NASH [11]. Therefore, the inhibition of NLRP3 is set to become a new point to target delaying the progression of NAFLD.

Exenatide (Exe, Byetta), the first glucagon-like peptide-1 (GLP-1) receptor agonist (GLP-1RA), has multiple biological effects, including improvements in glycemic control, lipid metabolism, and IR as well as preservation of *β* cell function [12]. Recent studies have shown that Exe also has extrapancreatic effects, such as weight loss and liver protection [13, 14]. Further research reveals that dipeptidyl peptidase-4 (DPP-4) inhibition by saxagliptin prevents inflammation and renal injury by targeting against the NLRP3/ASC inflammasome and that liraglutide pretreatment attenuates lipopolysaccharide-induced acute lung injury by inhibiting the NLRP3 inflammasome pathway in mice [15, 16]. However, the specific mechanism is not clear. GLP-1 has been proven to be effective in improving hepatocyte steatosis by inducing liver autophagy [17, 18]. Therefore, we hypothesized that Exe is involved in the improvement of NAFLD by targeting the NLRP3 inflammasome through the autophagy/mitophagy pathway. In this study, we selected the autophagy/mitophagy-related protein, NLRP3, and its downstream molecule IL-1*β*, to explore the liver-protective effect of Exe on HFD-induced NAFLD and diabetes in C57BL/6 mice.

## 2. Materials and Methods

### 2.1. Reagents

Exenatide (Amylin Pharmaceuticals Inc./Eli Lilly & Co.) and monoclonal anti-LC3A/B, anti-Beclin-1, anti-Parkin, anti-BCL2/adenovirus E1B 19 kDa protein-interacting protein 3-like (BNIP3L), anti-NLRP3, and anti-IL-1*β* antibodies were purchased from Cell Signaling Technology (USA). The anti-GADPH antibody was purchased from Abcam.

### 2.2. Animals

Thirty male 6- to 8-week-old C57BL/6 mice (18–20 g) were purchased from Vital River (Beijing, China). They were housed under a 12 h light/dark cycle with 50% humidity in a temperature-controlled setting. The mice were divided into a regular chow diet group (control group, *n* = 10) and a high-fat diet (the composition was as follows: 10% lard, 2.5% cholesterol, 1% bile salts, 20% sucrose, and 66.5% regular chow diet) group (HFD group, *n* = 20). The HFD group was used to induce fatty liver and diabetes. The mice in the HFD group were raised for 10 weeks and then provided with an intraperitoneal injection of 1% streptozotocin (STZ) solution (30 mg/kg). After 72 hours, a blood glucose meter was used to detect the FBG for two days continuously, and a random blood glucose > 16.7 mmol/L was used to indicate a successful diabetes model. At week 11, HFD mice were divided randomly into the NAFLD and diabetes model group (model group, *n* = 10) and the Exe treatment group (Exe group, *n* = 10).

The mice in the control and model groups were given an intraperitoneal injection of saline, and the Exe group was treated with an intraperitoneal injection of Exe (10 *μ*g/kg, bid) for four weeks. At the end of the experiment, 7 mice remained in the control and Exe groups, and 8 remained in the model group. The mice were then deeply anaesthetized with an intraperitoneal injection of 2% pentobarbital (60 mg/kg), and it was ensured that no response occurred after cornea stimulation. Blood samples were collected from the eyeballs. Livers were weighed immediately after sacrifice and frozen at −80°C for subsequent analysis. The study conformed to the principles of the Declaration of Helsinki, and all animal procedures were conducted in accordance with the National Institutes of Health's *Guide for the Care and Use of Laboratory Animals* (NIH Publications number 8023, revised 1978).

### 2.3. Biochemical Assays

Serum was collected by centrifugation of blood samples at 4000*g* for 15 min at 4°C. The blood glucose concentration was measured using the glucose oxidase method. The serum total cholesterol (TC), triglyceride (TG), and alanine transferase (ALT) levels were measured with kits (Nanjing Jiancheng, Jiangsu, China) according to the manufacturer's instructions.

### 2.4. Histopathology and Transmission Electron Microscopy

Liver specimen slices were processed according to a standard hematoxylin and eosin (H&E) staining technique, after which these tissues were observed for pathological changes under an optical microscope. Liver tissues were fixed in 2.5% glutaraldehyde. After being embedded, sectioned, and double-stained with uranyl acetate and lead citrate, images were captured with a transmission electron microscope (TEM) (JEOL, Tokyo, Japan).

### 2.5. Immunohistochemistry

Fixed liver tissue samples were embedded in paraffin and sectioned before the immunostaining assays using antibodies against NLRP3. Immunohistochemical staining was detected using a streptavidin-peroxidase complex.

### 2.6. Determination of Hepatic Lipid Metabolism and Oxidative Stress Injury Indicators

The liver homogenate was extracted, and the supernatant was measured. The liver tissue levels of TC, TG, and the liver injury indicator ALT were measured with kits (Nanjing Jiancheng, Jiangsu, China) according to the manufacturer's instructions. The serum and liver tissue levels of the lipid oxidative stress injury indicator malondialdehyde (MDA) were detected by an MDA assay kit using the thiobarbituric acid (TBA) method (Nanjing Jiancheng, Jiangsu, China), according to the manufacturer's instructions.

### 2.7. Western Blot Analysis

Liver proteins were harvested using RIPA buffer containing 1% protease inhibitor (PMSF, Beyotime, Shanghai, China). The phosphatase inhibitor (10%) was added when phosphorylated proteins were to be detected. Protein samples were size-separated on 8%–12% sodium dodecyl sulfate-polyacrylamide gels and then transferred onto polyvinylidene difluoride (PVDF) membranes (ISEQ00010 0.22 mm, Millipore, USA). Membranes were blocked with 5% nonfat milk for one hour and then incubated with primary antibodies against LC3A/B, Beclin-1, Parkin, BNIP3L, NLRP3, and IL-1*β* overnight at 4°C. After washing, the membrane was incubated with the secondary antibody for one hour. Chemiluminescent signals were developed with an ECL kit and detected with the ChemiDoc XRS gel documentation system (Bio-Rad, Hercules, CA). The protein bands were analyzed using Image Lab, and GADPH was used as an internal control.

### 2.8. Statistical Analysis

All values are expressed as mean ± SD. The statistical analysis was conducted with the SPSS 17.0 software. The comparison of multiple groups was performed using one-way analysis of variance (ANOVA). *p* < 0.05 was considered statistically significant.

## 3. Results

### 3.1. Exe Improves FBG and Serum Lipid Profiles

We successfully established a mouse model of NAFLD and diabetes by feeding HFD and by injecting STZ. As shown in Table 1, the FBG level in the model group was higher than that in the control group (*p* < 0.001), and Exe-treated mice showed a significantly decreased FBG after four weeks compared with the model group (*p* < 0.001). To investigate the relationship between Exe and the serum lipid profiles, the levels of the serum lipid profiles, including TC and TG, were examined. We found that the administration of Exe significantly decreased the serum TC and TG levels compared with the model group (*p* < 0.01 and *p* < 0.001, resp.) (Table 1).

### 3.2. Exe Decreases Body Weight and Liver Weight and Relieves Hepatic Lipid Accumulation

**Figure 1:**
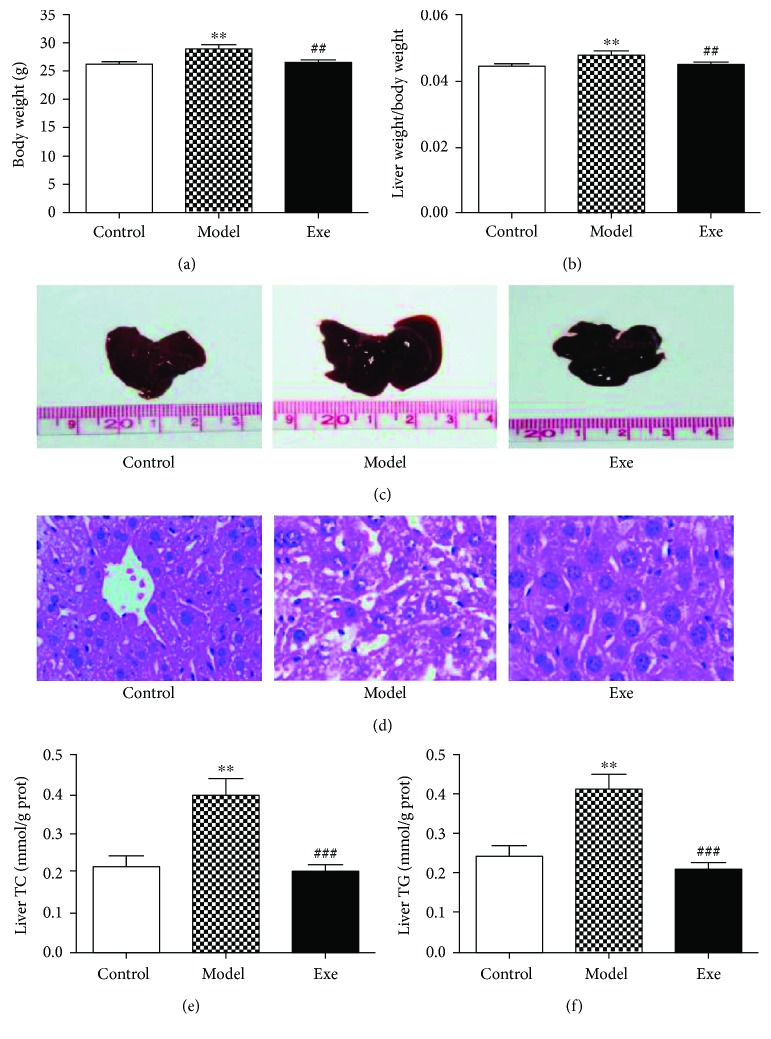
Effect of Exe on reducing body and liver weight and relieving hepatic lipid accumulation after four weeks of treatment: (a) body weight; (b) ratio of liver weight to body weight; (c) liver size; (d) representative photographs (400x) of H&E-stained liver sections. Liver TC (e) and TG (f) levels were determined. Data are expressed as mean ± SD (*n* = 7-8). ^∗∗^*p* < 0.01, model versus control group; ^##^*p* < 0.01 and ^###^*p* < 0.001, Exe versus model group. Exe: exenatide; TC: total cholesterol; TG: triglyceride.

Chronic treatment with Exe in model mice significantly reduced the body weight (*p* < 0.01, Figure 1(a)). We also determined the liver weights in all mice. As shown in Figure 1(b), the ratio of liver weight to body weight was significantly reduced after Exe treatment compared with that of the model group (*p* < 0.01). The liver size in the model group was larger than that in the control group, and it tended to be smaller after Exe treatment (Figure 1(c)). H&E staining showed that the number of lipid droplets in the livers increased significantly in the model group compared to the control group and decreased in the Exe group (Figure 1(d)). We then tested the TC and TG contents in the livers; they were significantly increased in the model group and dramatically decreased in the Exe group (both *p* < 0.001, Figures 1(e) and 1(f)).

### 3.3. Exe Attenuates Oxidative Stress and Liver Injury Indicators

**Figure 2:**
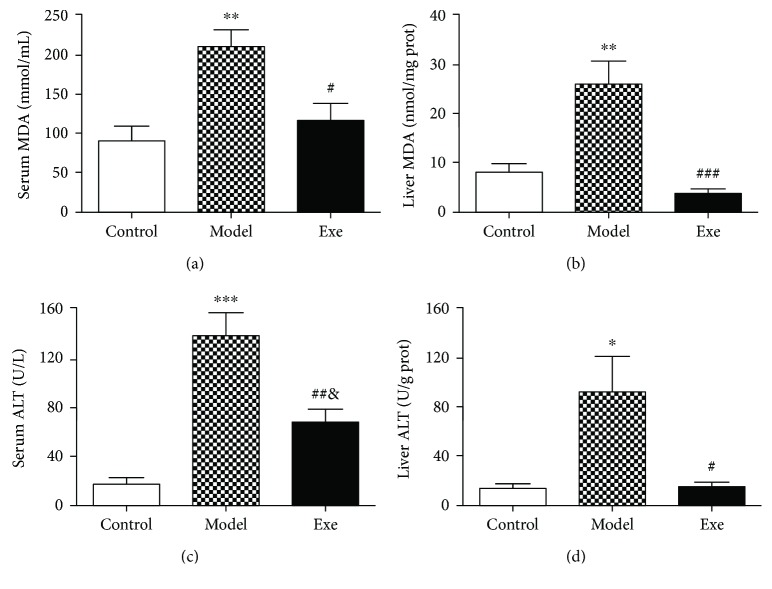
Exe attenuates oxidative stress and liver injury after four weeks of treatment: (a) serum MDA levels; (b) liver MDA levels; (c) serum ALT levels; (d) liver ALT levels. Data are expressed as mean ± SD (*n* = 7-8). ^∗^*p* < 0.05, ^∗∗^*p* < 0.01, and ^∗∗∗^*p* < 0.001, model versus control group; ^#^*p* < 0.01, ^##^*p* < 0.01, ^###^*p* < 0.001, and ^&^*p* < 0.05, Exe versus model group. Exe: exenatide; MDA: malondialdehyde; ALT: alanine aminotransferase.

The serum and liver lipid levels of the oxidative stress injury indicator, MDA, were tested. MDA increased greatly in the model group compared with the control group (both *p* < 0.01) and dramatically decreased in the Exe group (*p* < 0.05 and *p* < 0.001, resp.) (Figures 2(a) and 2(b)). Serum levels of the liver injury indicator, ALT, were significantly increased in the model group compared to the control group (*p* < 0.001) and decreased sharply after Exe treatment (*p* < 0.01, Figure 2(c)). ALT levels in liver tissues were similar to those in serum (Figure 2(d)), indicating that treatment with Exe could inhibit liver lipid oxidative stress injury.

### 3.4. Exe Induces Liver Autophagy/Mitophagy and Inhibits the NLRP3 Inflammasome

**Figure 3:**
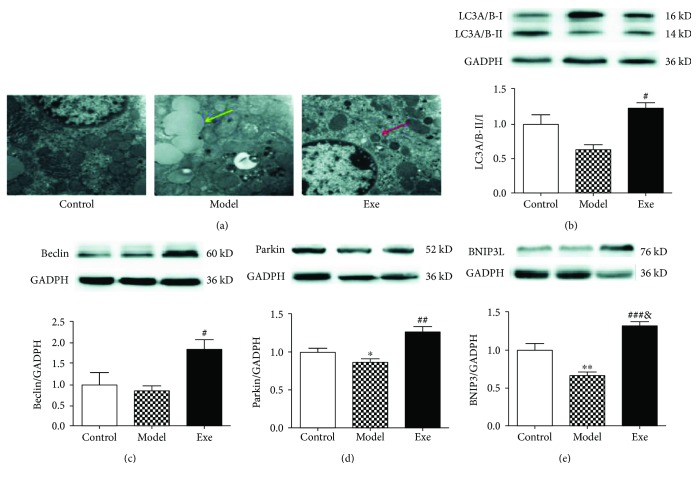
Exe enhances liver autophagy/mitophagy and inhibits the NLRP3 inflammasome. (a) Representative TEM images (20000x) from TEM showing characteristic autophagosomes (red arrow) and lipid droplets (yellow arrow) in livers. (b, c, d, e) Effects of Exe on LC3A/B, Beclin-1, Parkin, and BNIP3L in the livers as detected by Western blot. GAPDH served as the loading control. Representative Western blot images from each group were shown. Data are expressed as mean ± SD (*n* = 5-6). ^∗^*p* < 0.05, ^∗∗^*p* < 0.01, model versus control group; ^#^*p* < 0.01, ^##^*p* < 0.01, and ^###^*p* < 0.01, Exe versus model group; ^&^*p* < 0.05, Exe versus control group. Exe: exenatide; TEM: transmission electron microscope; GADPH: glyceraldehyde 3-phosphate dehydrogenase.

**Figure 4:**
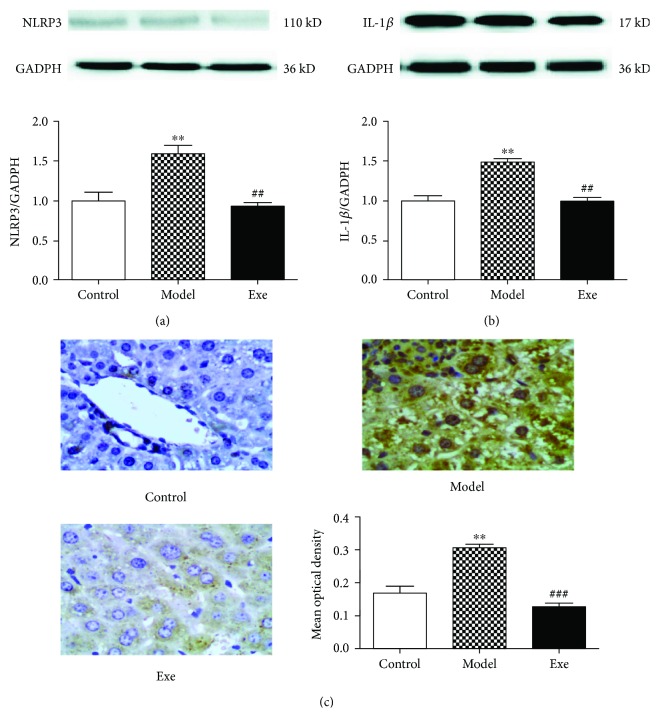
Effect of Exe on the NLRP3 inflammasome and IL-1*β* after four weeks of treatment. Effect of Exe on the expression of (a) NLRP3 and (b) IL-1*β* in the livers, as determined by Western blot. (c) The expression of NLRP3 in liver tissues, as shown by immunohistochemical staining of liver tissues. The positive staining for NLRP3 expression appears as brown-yellow granules. Representative Western blot and immunohistochemical images from each group were shown. Data are expressed as mean ± SD (*n* = 5-6). ^∗∗^*p* < 0.01, model versus control group; ^##^*p* < 0.01 and ^###^*p* < 0.01, Exe versus model group. Exe: exenatide; GADPH: glyceraldehyde 3-phosphate dehydrogenase.

We then examined the autophagic changes in all groups. TEM imaging showed that Exe treatment increased the number of autophagosomes (red arrow) and lipid droplets (yellow arrow) in model mice (Figure 3(a)). Western blot was used to determine the expression of autophagy/mitophagy-associated proteins. As shown in Figures 3(b)–3(e), the ratio of LC3A/B-II and LC3A/B-I and the expression of Beclin-1, Parkin, and BNIP3L were more pronounced in the Exe group. To detect the relationship between autophagy/mitophagy and inflammasomes, the NLRP3 inflammasome and its downstream molecule IL-1*β* were also examined. As shown in Figures 4(a) and 4(b), the expression of NLRP3 and IL-1*β* was strongly increased in the model group and distinctly attenuated in the Exe group. Histologic analyses of the tissues further confirmed that Exe downregulates the expression of NLRP3 in the livers (Figure 4(c)). In addition, it was observed that the expression of NLRP3 increased significantly in the model group (Figure 4(c)), where it mainly appeared in the cytoplasm. These results indicated that treatment with Exe may inhibit the NLRP3 inflammasome by inducing autophagy/mitophagy.

## 4. Discussion

T2DM and NAFLD have become worldwide health concerns. Hepatic fat accumulation and mitochondrial dysfunction are important common links in the pathogenesis of T2DM and NAFLD [19]. T2DM accelerates the progression of NAFLD to NASH. The NLRP3 inflammasome is considered a key factor for the development of NASH induced by hyperlipidemia. Thus, regulating the NLRP3 inflammasome may provide a potential mechanism for NAFLD treatment. ROS from mitochondria is the key signal for activating the NLRP3 inflammasome [9]. A previous study showed that autophagic flux attenuates the activation of the NLRP3 inflammasome pathway in macrophages [20]. Therefore, the promotion of autophagy may theoretically inhibit the damage caused by the NLRP3 inflammasome. In the present study, we first demonstrated that Exe inhibited oxidative stress injury response and then showed that the activation of the NLRP3 inflammasome, which was mediated by the autophagy/mitophagy pathway, delayed the progression of HFD-induced NAFLD.

In these experiments, we successfully established a mouse model of NAFLD and diabetes using HFD and streptozocin [21, 22]. In our study, we found that body weight was significantly decreased in Exe-treated mice. Previous studies have confirmed that there are many mechanisms for Exe-induced weight loss, including increased central satiety, retarded gastric emptying, improved insulin sensitivity of peripheral tissues, and the induction of the browning in white adipose tissue and thermogenesis in brown adipose tissue. These effects are the basis for the weight loss of Exe. However, Exe is also good for systemic metabolism, because the levels of FBG, TC, and TG in serum decreased significantly after Exe treatment. The reason for these results may be the effect of Exe on promoting insulin secretion, inhibiting glucagon secretion, and improving islet beta cell proliferation, regeneration, and differentiation [23]. All of these may ultimately improve IR in peripheral tissues. Of course, the effect of Exe on weight loss and the improvement of systemic metabolism may be among the reasons for the improvement in fatty liver.

The liver itself is a predominant machine for keeping weight under control and lipid metabolism balance by a complicated set of biochemical pathways, being both a fat-burning organ and a fat-pumping organ. In this study, we observed a remarkable reduction of liver weight after Exe treatment both before and after weight adjustment. The liver size and the ratio of liver weight to body weight both decreased dramatically after Exe treatment. Furthermore, the number of lipid droplets in the liver exhibited a sharp rise in the model group of mice and decreased markedly after Exe intervention. The liver TC and TG contents were also dramatically decreased after four weeks of Exe treatment. These results show that Exe might have a potential benefit to the liver, as confirmed by our previous clinical study [14]. Gupta et al. confirmed that the GLP-1R is present in human hepatocytes, thus suggesting a direct effect of GLP-1 on the reduction of hepatic steatosis [24]. In vitro studies further showed that GLP-1 could improve the insulin sensitivity and regulate lipid metabolism-related genes in hepatocytes by acting via the GLP-1R in hepatocytes [25, 26]. Further, GLP-1 can clear excessive lipid accumulation in the liver by enhancing hepatic autophagy and increasing the expression of fatty acid transporter protein [27, 28]. However, the limitation of this study is that liver lipid metabolism-related enzymes, products, and transporters and other related indicators were not examined, all of which require further validation.

Importantly, liver histopathology showed obvious hepatic steatosis and few cells infiltrating into the liver lobules. We measured MDA, an end product of lipid peroxidation, to evaluate the oxidative stress. In the present study, the MDA content was significantly increased in the model mice. We also tested the liver injury marker, ALT, to assess the extent of liver damage. The ALT levels were dramatically increased in the model group. The microstructural changes of hepatocytes were confirmed by TEM, which showed that the mitochondria were obviously swollen and damaged. In NAFLD and diabetes, the absence of mitochondrial autophagic clearance and redundant mitochondrial ROS lead to aggravated oxidative stress injury and the induction of inflammation, which may be the cause of hepatocyte damage in the liver. In contrast, the above indicators were obviously improved after four weeks of Exe treatment.

**Figure 5:**
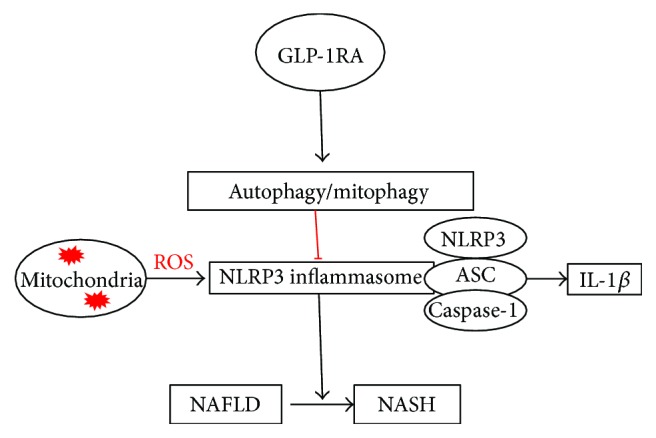
Summary of working thesis of the study. The hypothesis arising from the data.

The activation of the NLRP3 inflammasome plays a crucial role in the inflammatory progression of NAFLD [9, 29]. Therefore, we evaluated the expression of the inflammation indexes NLRP3 inflammasome and IL-1*β* at the protein level to determine the effect of Exe on inflammation. The increased levels of NLRP3 and IL-1*β* in the model mice were obviously alleviated by Exe. Further histologic analyses also confirmed that Exe downregulated the expression of NLRP3 in the model mice. Moreover, we found that autophagy/mitophagy was suppressed in the livers of HFD-induced NAFLD and diabetic mice. The results indicated that the levels of LC3 and Beclin-1, which are key proteins required for autophagosome formation, and of Parkin and BNIP3L, which are important proteins in the mitophagy pathway, were significantly decreased in the model mice. Nevertheless, the expression of LC3A/B-II/I, Beclin-1, Parkin, and BNIP3L and the number of autophagosomes in the Exe group were notably increased. Studies have suggested that enhancing autophagy/mitophagy will remove the damaged mitochondria and thereby prevent the ROS-induced inflammatory response [9]. Therefore, our results primarily validated our hypothesis that Exe could inhibit the NLRP3 inflammasome by enhancing autophagy/mitophagy and depressing oxidative stress, which will further delay hepatic inflammation progression (Figure 5).

There exists evidence to support our theory that GLP-1 or DPP-4 inhibitors can suppress the NLRP3 inflammasome, but the specific mechanisms have yet to be elucidated [15, 30, 31]. Of course, our present study only preliminarily verifies the potential hepatoprotective effect of Exe at the animal level, and the mechanism needs to be explored at the cellular level. In addition, whether Exe has a direct effect on the NLRP3 inflammasome needs to be investigated.

In conclusion, the present study demonstrated that Exe could eliminate the excessive damaged mitochondria, thereby reducing oxidative stress injury and then inhibiting NLRP3 inflammasome activation via the mitophagy pathway. Ultimately, Exe exerted an early protective effect on the liver in NAFLD and diabetes mice, considering the delay in NAFLD progression. Our results provide new insights and more theoretical support for the clinical application of Exe to achieve effective interventions and liver benefits.

## Figures and Tables

**Table 1:** Effects of Exe on FBG and serum lipid profiles after four weeks of treatment.

	Control	Model	Exe
FBG (mmol/L)	8.92 ± 1.87	15.62 ± 3.66^∗∗∗^	9.28 ± 1.74^###^
TC (mmol/L)	9.12 ± 3.93	11.58 ± 4.07	5.25 ± 3.36^##^
TG (mmol/L)	3.73 ± 1.63	4.97 ± 1.26	1.66 ± 0.62^###&^

Data are expressed as mean ± SD (*n* = 7-8). ^∗∗∗^*p* < 0.001, model versus control group; ^##^*p* < 0.01 and ^###^*p* < 0.001, Exe versus model group; ^&^*p* < 0.01, Exe versus control group. Exe: exenatide; FBG: fasting blood glucose; TC: total cholesterol; TG: triglyceride.
